# Identification of organic pigments in tattoo inks and permanent make-up using laser desorption ionisation mass spectrometry

**DOI:** 10.12688/f1000research.13035.2

**Published:** 2018-01-08

**Authors:** Markus Niederer, Urs Hauri, Lydia Kroll, Christopher Hohl

**Affiliations:** 1 State Laboratory Basel-City, Basel, 4056, Switzerland

**Keywords:** tattoo inks, PMUs, colour pigments, MALDI-TOF

## Abstract

Nowadays, about 12% of the European and 20% of the US population are tattooed. Rising concerns regarding consumer safety, led to legal restrictions on tattoo and permanent make-up (PMU) inks. Restrictions also include bans on certain colourants. Both ink types use organic pigments for colour-giving, plus inorganic pigments for white and black and colour tones. Pigments are only sparingly soluble in common solvents and occur as suspended particles in the ink matrix. Their detection and identification therefore pose a major challenge for laboratories involved in monitoring the legal compliance of tattoo inks and PMU. We overcame this challenge by developing a direct laser desorption ionisation time-of-flight mass spectrometry method, which included an easy sample clean up. The method proved to be capable of detecting and identifying organic pigments in almost all of the tested ink samples. Method validation and routine deployment during market surveys showed the method to be fit for purpose. Pigment screening of 396 tattoo inks and 55 PMU taken from the Swiss market between 2009 and 2017 lead to the following conclusions: Pigment variety is much greater in tattoo inks (18) than in PMU (10); four prohibited pigments (Pigment Green 7, Pigment Red 122, Pigment Violet 19 and 23) were found in both ink types; for PMU, these four pigments made up 12% of the pigment findings, compared to 32% for tattoo inks. Therefore, legal compliance of PMU was at a higher level. A comparison of pigments found with those declared on tattoo ink labels clearly showed that banned pigments are rarely declared, but rather masked by listing non present legal pigments and label forging; therefore, highlighting the urgency of widespread market controls.

## Introduction

Over the last two decades, tattoos have become increasingly popular among younger people, with estimates stating that 12% of the European and more than 20% of the US population are tattooed ^1^. These figures, the widespread on-line sales of tattoo related products, and the invasive techniques used during tattooing - where inks are injected into the skin’s dermis - have led to serious concerns on the safety of tattoo inks and permanent make-up (PMU). This finally compelled several European nations to initiate specific legal regulations, which came into effect during the last ten years and are all based on the European Resolution (ResAP 2008) ^2, 3^. For colourants, namely pigments, being the ingredients, which give tattoo inks and PMU the desired effect, regulations are summarised as follows: a ban on colourants that can form aromatic amines under reductive cleavage, a negative list for specific colourants and a ban for colourants with restricted use in cosmetics. Despite this welcome development, safety awareness in this field is still not adequate compared to cosmetics, which have been subjected to stringent regulations for decades, even though their application forms are solely non-invasive. Regulations for cosmetics also require manufacturers to evaluate the consumer safety of their cosmetic products and ingredients. In contrast to this, none of the ingredients used in tattoo inks have ever been tested to ensure their health safety when injected into the skin. Furthermore, the generalised assumption that pigments as insoluble colourants pose no health risk, does not hold true in the dermis. Pigment fading and transport of organic pigments from the skin to regional lymph nodes have been well documented for tattoos ^4, 5^. Studies have reported that diarylide pigments degrade under sunlight forming a variety of products, some of which are known to be toxic or carcinogenic ^6^. This finding has yet to be incorporated into regulations.

Pigments are at best only sparingly soluble in common solvents, which narrow the choice of suitable analytical techniques. So far the identification of tattoo pigments in inks has mainly been carried out using Raman as well as Fourier transform infrared (FT-IR) spectroscopy ^7, 8^. Raman spectroscopy has also been used for matching the profile of tattoo inks with dermatome shave biopsies from patients with allergic reactions ^9^. Another recent paper describes the successful use of pyrolysis gas chromatography (py-GC/MS) ^10^. While FT-IR and Raman Spectroscopy are very suitable for the identification of single pigments, their successful use for pigment mixtures, often present in tattoo inks, has yet to be proven. Direct laser desorption or matrix assisted laser desorption ionisation time-of-flight mass spectrometry (LDI-TOF-MS or MALDI-TOF–MS) has been reported for identifying pigments in automotive coatings ^11^ or art work ^12– 14^. In this article, we present a validated LDI-TOF-MS approach for the identification of pigments in tattoo inks and PMU. Repeated market surveys in Switzerland showed the method to be fit for purpose.

## Methods

### Chemicals

As reference materials, more than 150 individual pigments from different producers (mainly BASF (Basel, Switzerland), Clariant (Muttenz, Switzerland) supplied by Omya (Oftringen, Switzerland), Kremer Pigmente (Aichstetten, Germany) and Sigma–Aldrich (Buchs, Switzerland)) were collected over the past years. Mass calibration was performed with the following dyes all from Sigma-Aldrich: Crystal Violet (C.I. 42555), Basic Blue 7 (C.I. 42595), Ethyl violet (C.I. 42600), Basic Blue 11 (C.I. 44040), Basic red 2 (C.I. 50240), Basic Blue 17 (C.I. 52040). MALDI matrix 2,5-Dihydroxybenzoic acid (DHB, 99%) was purchased from Sigma–Aldrich. Ethanol (EtOH) and methanol (MeOH), both analytical grades, were from J.T. Baker (Center Valley, USA).

### Suspensions and solutions

Stock suspensions and/or solutions of reference pigments in MeOH (1 mg mL ^-1^) were individually prepared. For mass calibration, a mixture containing six pigments at levels of 50 μg mL ^-1^ for C.I. 42555, C.I. 42595 and C.I. 42600, of 250 μg mL ^-1^ for C.I. 50240 and C.I. 52040 and of 100 μg mL ^-1^ for C.I. 44040 was prepared by mixing homogenous aliquots of each stock suspension and diluting with MeOH.

### Samples and sample preparation

Tattoo inks and PMU were taken from the Swiss market over the last eight years. An overview of the analysed samples is given in Table 1.

Ink samples were shaken intensely by hand for about one minute. Then, about 25 µL (20–30 mg) of the suspension or 20 mg of PMU were weighed into a tared 2 mL-tube, mixed with 1 mL of EtOH, sonicated (5 min, 25°C) and centrifuged (5 min, 15000 rpm). The supernatant was removed and the residue containing the colour pigments was diluted with 1 mL of MeOH. After vortexing for some seconds, 1 µL duplicates of the homogenous suspension were immediately spotted onto a steel target plate, air dried at room temperature and used for LDI-TOF-MS.

### Pigment verification with standard addition

20 µL of the methanolic ink suspension were transferred to a 96-Well reaction plate (MicroAmp Optical, Applied Biosystems) and mixed with 10 – 50 µL of the appropriate reference pigment suspension (10 mg mL ^-1^ MeOH). One microliter of the homogenous suspension was used for LDI-TOF-MS.

### Improvement of detection using DHB as a matrix

20 µL of the methanolic ink suspension were transferred to the reaction plate, mixed with the same volume of DHB (10 mg mL ^-1^ MeOH) and 1 µL of the homogenous suspension was used for MALDI-TOF-MS.

### MALDI-TOF-MS

Pigment mass spectra were obtained using a MALDI-TOF Mass Spectrometry Axima™ Confidence machine (Shimadzu-Biotech Corp., Kyoto, Japan) with detection in the reflectron mode with pulsed extraction (optimised at 450 Da) at a N _2_-laser frequency of 50 Hz and within a mass range from 50 to 2000 Da. The transmission of the laser power was in the range of 60 – 100 units (33 – 55%). The target plate was scanned by the laser (diameter of 30 μm) in the regular rectangular mode and serpentine style (1000 × 1000 μm, spacing 166.666 μm and 49 points). A minimum of 20 laser shots was accumulated per profile and for each sample a total of 50 mass profiles was averaged and processed using Launchpad™ version 2.9.3 software (Shimadzu-Biotech Corp., Kyoto, Japan). This software was also used for peak processing of all raw spectra with the following settings: the advanced scenario was chosen from the parent peak clean up menu, peak width was set to 3 channels, smoothing filter width to 2 channels, baseline filter width to 10 channels, and the threshold apex was chosen as the peak detection method. The threshold apex peak detection was set as a dynamic type and the offset was set to 0.300 mV with a response factor of 1.0. The processed spectra were exported as peak lists with m/z values for each peak and signal intensity in the ASCII format. Calibration was conducted for each target plate using spectra of the reference standard mixture consisting of the following exact masses: 271.1137, 315.1609, 372.2439, 422.2596, 456.3378 and 478.3222 amu.

### Identification of pigments with NIST library software

Exported ASCII files from MALDI-TOF-MS were converted to NIST software compatible peak lists with a mass resolution of 1 D (*.msp), and the identification of pigments was carried out with our home-made mass spectra library using the software of NIST 2.0 (Standard Reference Data Program of the National Institute of Standards and Technology, USA).

### HPLC

More or less soluble colourants detected by LDI-TOF-MS were occasionally confirmed by high performance liquid chromatography (HPLC). Approximately 5 mg of tattoo or PMU samples were extracted with different solvents, starting with N,N-Dimethylformamide, 1-Chloronaphthalene and N-Methylpyrrolidone, 1 mL each. Coloured extracts were centrifuged at 12’000 g, filtered with 0.45 µm PTFE syringe filters and analysed with Ion Pair Reversed Phase HPLC with Ultraviolet Diode Array (UV/DAD) detection under the following conditions: Kromasil-column C18, 5 µm, 150 × 2 mm (30°C); eluent A: aqueous solution of dodecyltrimethylammonium bromide (3 g/L) and ammonium bromide (1 g/L), eluent B: ethanolic solution of dodecyltrimethylammonium bromide (3 g/L) and ammonium bromide (1 g/L); run time = 30 minutes; flow rate = 0.35 mL/min; gradient conditions: 0 min 45% eluent B, 2 min 55% eluent B, 10 min 65% eluent B, 20 min 100% eluent B, 24 min 100% eluent B, 24.1 min 45% eluent B.

## Results and discussion

Over the last eight years, more than 150 individual colour pigments and about 450 commercial tattoo ink or PMU samples from market surveys were subjected to LDI- or MALDI-TOF-MS analyses. Mass profiles were recorded either from pigments of different producers in order to build a home-made reference spectrum library or from samples taken during market surveys. From these former investigations we derived the following conclusions: LDI- or MALDI-TOF-MS is an excellent and rapid method for the detection and identification of organic colour pigments, but not for inorganic ones. For the most part of the analysed pigments, the ionisation can be performed without added matrix (LDI-TOF-MS), because the pigments themselves function as chromophores absorbing the laser beam ^14^.

### Method overview

After clean-up, samples were screened with LDI-TOF-MS using the NIST program with our pigment library for identification. Tentatively identified pigments were then confirmed by comparing the high-resolution mass spectra of the native and the reference spiked sample. As a further check, the resulting colour of found pigments had to give the sample colour. Whenever the results of the analysis did not match with the colour of the samples, the ionisation was optimised by changing the laser power and/or adding DHB as a matrix to the sample.

### Effect of ethanolic sample clean-up

**Figure 1.**
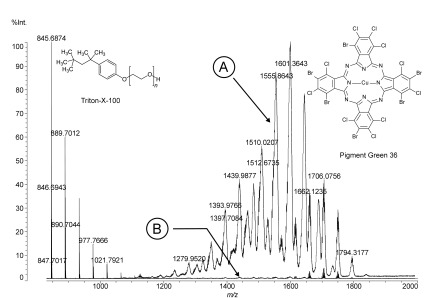
High resolution mass spectra of Pigment Green 36 (C.I. 74265) without any discrimination effect ( **A**) and affected by the surfactant Triton X-100 ( **B**).

Modern tattoo inks not only contain pigments but also binders, solvents, surfactants, preservatives and thickening agents ^15^. Surfactants adsorb to the surface of pigment agglomerates and decrease the surface tension of the solvent. This eliminates residing air bubbles and improves particle coating with other additives. Adsorbed surfactants, however, can interfere with analysis; spiking C.I. 74265 (pigment green 36) with the non-ionic tenside Triton X-100 leads to a nearly complete suppression of mass signals ( Figure 1). This problem was met by washing samples with EtOH before LDI-TOF-MS, which proved to be effective in most cases ^16^.

### Screening of samples with NIST software

**Figure 2.**
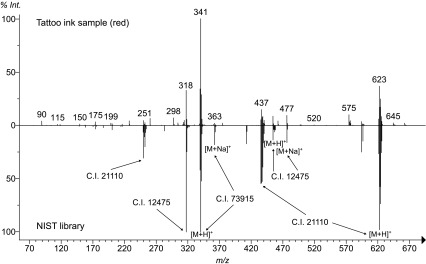
Head to tail plot of three pigments identified in the mass spectra of a red tattoo ink sample by NIST software: C.I.73915 (Pigment Red 122, R. Match = 878), C.I.21110 (Pigment Orange 13, R. Match = 833) and C.I.12475 (Pigment Red 170, R. Match = 780).

With no commercial library of pigments available, we made our own library by exporting ASCII files of the mass spectra of analysed reference pigments to NIST compatible unit resolution mass peak lists. We preferred the NIST software for screening samples because its reversed match algorithm (R. Match) allows the simultaneous identification of several pigments in mixtures. This advantage is demonstrated for a red tattoo ink in Figure 2. Although the three existing pigments have overlapping ion clusters, all could be identified by their characteristic ions: C.I. 73915 (P.R. 122) with the ions [M+H] ^+^ (m/z 341) and the sodium adduct [M+Na] ^+^ (m/z 363), C.I. 21110 (P.O. 13) with the molecular ion [M+H] ^+^ (m/z 623) and the two fragment ion clusters at m/z 437 and m/z 251 and C.I. 12475 (P.R. 170) with the ions [M+H] ^+^ (m/z 455), [M+Na] ^+^ (m/z 477) and the fragment ion m/z 318. As a further advantage of the NIST software compound specific information such as structural formula, molecular mass, synonyms, colour and CAS-no. can be added to the mass spectra library, and therefore also be used as search criterions. In the case of unknown mass spectra, the software can also extend the search to commercial GC-MS mass libraries (e.g. NIST main library).

### Verification of pigments

**Figure 3.**
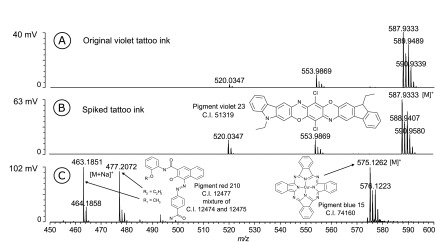
Verification of the prohibited violet pigment C.I. 51319 in a tattoo ink sample by high resolution mass spectra ( **A**) and standard addition ( **B**), as well as the exclusion of a legal red (C.I. 12477) and blue (C.I. 74160) reference pigment ( **C**) in the sample.

Standard addition and high-resolution mass spectrometry were used for the verification of particularly interesting pigments tentatively identified by NIST. As a quality control standard addition also allows checking for possible discriminations caused by the sample matrix. Verification is shown for a violet coloured tattoo ink containing the prohibited C.I. 51319 (P.V. 23), which was not declared ( Figure 3). After being tentatively identified in a first run, spiking the sample with C.I. 51319 ( Figure 3B) showed the same typical molecular ion cluster [M] ^+^ (m/z 588) and two fragment ion clusters with m/z 554 and m/z 520 as in the original sample ( Figure 3A). Instead of C.I. 51319, the product label listed two legal pigments red C.I.12477 (P.R. 210) and blue C.I.74160 (P.B. 15) both non-present in the ink. LDI spectra did not reveal their characteristic ions [M+Na] ^+^ (m/z 463) and [M+Na] ^+^ (m/z 477) for the red pigment (a mixture of C.I. 12474 and 12475) and the ion cluster [M+H] ^+^ (m/z 575) of the blue one ( Figure 3C). This demonstrates a typical case of label forging for tattoo inks.

### Signal enhancement using DHB as matrix

**Figure 4.**
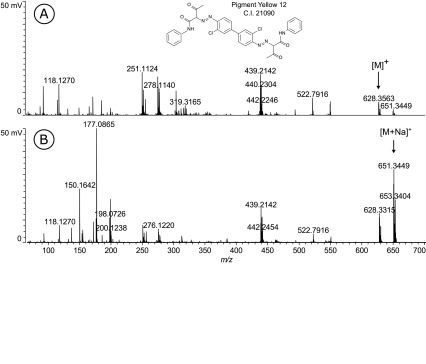
LDI mass spectra of pigment Yellow 12 (C.I. 21090) without matrix ( **A**) and MALDI spectra of the same pigment with DHB as a matrix ( **B**).

In the case of some 3,3’-dichlorobenzidine based diazodiaryl-pigments, only weak molecular ion mass signals ([M] ^+^, [M+H] ^+^, [M+Na] ^+^) but more pronounced fragmentation were observed. In order to enhance molecular mass signals, three of the most often used MALDI- matrices, dihydroxybenzoic acid (DHB), α-cyano-4-hydroxycinnamic acid (CCA), and sinapic acid (SA), were added to ink samples containing dichlorobenzidine based pigments. It turned out that all three had a positive effect, with DHB showing the best signal enhancement. The effect of DHB on the mass spectra of yellow pigment C.I. 21090 is shown in Figure 4. With DHB the signal intensity of the molecular ion [M] ^+^ (m/z 628) was twice that of the sodium adduct [M+Na] ^+^ (m/z 651), even twelve times as high as without matrix supplement.

### Method validation

Because the identification of pigments by LDI-TOF-MS is a qualitative method, validation was only performed for mass resolution, accuracy of pigment identification, a rough estimation of limits of detection and the precision of characteristic mass intensity.

At the mass of 450 amu, a mass resolution of R = M/ΔM = 6000 (full width at half maximum, FWHM) was achieved. The resolution quality depends on the laser power and is not the same for all pigments. Generally, mass resolution decreases with higher laser power and a shift to a higher mass of up to 0.1 u can be observed.

Accuracy of pigment identification in the screening mode (NIST, without sample washing and spiking) was tested with 48 different tattoo ink samples, their composition being unknown to the analyst. The results were checked for conformity using trustworthy declarations and/or additional in-house HPLC analysis of more or less soluble colourants. In 44 of the 48 samples (92%) all organic pigments were identified correctly (up to four in the same sample). Incomplete detection was found in two inks where the banned orange pigment C.I. 12075 remained undetected due to its weak signals in the mass spectra. The resulting poor library search matches did not give sufficient evidence for a positive identification. With the characteristic ion [M+H] ^+^ (m/z 339), this pigment is usually well detectable. Therefore, a pigment concentration near the limit of detection is the most likely explanation for the weak signal. For the two other samples, results of LDI-TOF-MS were not consistent with those of HPLC-analysis: in the first case, C.I. 21110 was identified with LDI-TOF-MS, whereas HPLC-analysis gave C.I. 21115. In the second case C.I. 21100 was mistaken for C.I. 21095 because the characteristic mass signals of C.I. 21100 (m/z 522 and 550) were quenched.

The limits of detection (LOD) in a blue tattoo ink were estimated by standard addition with two red pigments, of which one was a well (C.I. 56110) and the other a poorly ionisable one (C.I. 14720). A correct identification of the pigment specific mass clusters by the NIST library was possible between sample concentrations of 1% (w/w) for the well ionisable pigment and of 10 – 20% (w/w) for the other one (C.I. 14720). As pigments are added to tattoo inks in the percent range, screening with LDI-TOF-MS proved to have a suitable LOD.

Nevertheless, we have to consider that sometimes the LOD can also be affected by discrimination effects of tensides or inorganic pigments, such as carbon black (C.I. 77266) or TiO _2_ (C.I. 77891, white), present in the sample ^16^. Whereas tensides can be easily removed by EtOH the separation of the two inorganic pigments remain an unresolved problem up to this date. However, with the application of a higher laser energy and colour plausibility checks, negative identification results due to matrix effects could be eliminated for all forbidden organic pigments.

Precision was determined as repeatability with ten determinations of the characteristic mass intensity of two pigments (C.I. 21108, C.I. 74260) and two different inks containing C.I.74160 and C.I.12475, respectively. The pigment specific relative standard deviations were in a range of 4% to 13%, which are by far sufficient for identification purposes. This good reproducibility is in contrast to common experience with MALDI-TOF-MS as inhomogeneous crystallisation of the matrix promotes the building of so called “hot-spots” with locally higher ionisation signals ^17^. Obviously, the dispersion of the pigments spotted on the target plate using MeOH instead of a matrix seems to give a more homogenous distribution of the analytes. Good reproducibility was probably also due to laser scanning in the serpentine style over the whole sample with 20 accumulated laser shots per profile which evens out some inhomogeneity.

### Market survey

Over the last eight years we used LDI- or MALDI-TOF-MS for market survey purposes in Switzerland. Samples were randomly but also risk based collected from tattooing and PMU studios and from different importers. Table 1 gives an overview of the organic pigments identified in tattoo ink and PMU samples. Data obtained show that Pigment Blue 15 (C.I. 74160) was the most often found colourant followed by the prohibited Pigment Green 7 (C.I. 74260). All prohibited pigments, especially Pigment Green 7, were more frequently found in inks (3 – 13%) than in PMU (2 – 4%). In contrast to detected legal pigments where only 7% were missing on product labels of the samples, the prohibited pigments were often not declared (68%), for details see 18. This clearly gives evidence that label forging is widespread. This fraud considers pigments that are banned due to toxicological concerns, showcasing the urgency for widespread market controls.

**Table 1.** Proportions of the most frequently identified pigments (≥ 2%) in about 450 products of Swiss market surveys between 2009 and 2017. Prohibited colours are marked with an *.

Pigment	Name	Total samples (n=451)	Tattoo inks (n=396)	Permanent make-up (n=55)
C.I. 74160	Pigment Blue 15	27%	28%	16%
C.I. 74260 *	Pigment Green 7	12%	13%	4%
C.I. 12475	Pigment Red 170	10%	11%	4%
C.I. 56110	Pigment Red 254	9%	8%	16%
C.I. 73915 *	Pigment Red 122	8%	8%	4%
C.I. 561170	Pigment Orange 73	8%	6%	16%
C.I. 51319 *	Pigment Violet 23	8%	8%	2%
C.I. 11741	Pigment Yellow 74	5%	6%	-
C.I. 21095	Pigment Yellow 14	5%	6%	-
C.I. 21110	Pigment Orange 13	5%	6%	-
C.I. 12477	Pigment Red 210	5%	5%	-
C.I. 56300	Pigment Yellow 138	4%	5%	-
C.I. 12490	Pigment Red 5	4%	2%	24%
C.I. 74265	Pigment Green 36	4%	5%	-
C.I. 51345	Pigment Violet 37	3%	4%	-
C.I. 11767	Pigment Yellow 97	3%	3%	4%
C.I. 73900 *	Pigment Violet 19	3%	3%	2%
C.I. 11740	Pigment Yellow 65	3%	3%	-

MALDI raw data are provided as MASTER_RUN-, CAL-, COR-, LBL-, RAW, RUN, STATS- and UAP- files using Launchpad™ version 2.9.3 software (Shimadzu-Biotech Corp., Kyoto). The data include the raw high resolution spectra of Figures 1, 3 and 4 Click here for additional data file. Copyright: © 2018 Niederer M et al. 2018 Data associated with the article are available under the terms of the Creative Commons Zero "No rights reserved" data waiver (CC0 1.0 Public domain dedication).

MALDI raw data are provided as TXT-files in ASCII-format using Launchpad™ version 2.9.3 software (Shimadzu-Biotech Corp., Kyoto). The data include the high resolution raw spectra of Figures 1, 3 and 4 Click here for additional data file. Copyright: © 2018 Niederer M et al. 2018 Data associated with the article are available under the terms of the Creative Commons Zero "No rights reserved" data waiver (CC0 1.0 Public domain dedication).

NIST spectrum peak lists are provided as MSP-files in ASCII-format with a mass resolution of 1 D (msp) using the software of NIST 2.0 (USA). The data include the NIST spectra of Figure 2, Table 1 and method validation (HPLC verification) Click here for additional data file. Copyright: © 2018 Niederer M et al. 2018 Data associated with the article are available under the terms of the Creative Commons Zero "No rights reserved" data waiver (CC0 1.0 Public domain dedication).

Intermediary data of Table 1 and a list of suppliers of pigments of Table 1 are provided as Excel-files (XLS). The data include sample-nr, declaration of prohibited and legal pigments and reversed match values of pigments by NIST-software (Rfit) Click here for additional data file. Copyright: © 2018 Niederer M et al. 2018 Data associated with the article are available under the terms of the Creative Commons Zero "No rights reserved" data waiver (CC0 1.0 Public domain dedication).

An example of the construction of our home-made spectrum library using the NIST software 2.0 (USA) is shown as JPG-file. The data include compound name, structural formula, molecular mass, CAS-Nr., analysis date, intensity of the main spectrum peaks and synonyms Click here for additional data file. Copyright: © 2018 Niederer M et al. 2018 Data associated with the article are available under the terms of the Creative Commons Zero "No rights reserved" data waiver (CC0 1.0 Public domain dedication).

## Conclusion

To our knowledge, no study reported a LDI-TOF-MS method for the identification of colour pigments in tattoo inks, PMU or cosmetic products. Method validation and using the LDI-TOF-MS method for market surveys demonstrated that the method described is fit for purpose. Therefore, LDI-TOF-MS is a further powerful tool particularly in combination with py-GC/MS ^10^, HPLC or ultraviolet–visible spectroscopy ^18^ for enforcing legal restrictions on pigments in tattoo inks and PMU. It should be kept in mind, however, that the presented method gives no information on the levels of the detected organic pigments. For this, further studies would be necessary.

## Data availability

The data referenced by this article are under copyright with the following copyright statement: Copyright: © 2018 Niederer M et al.

Data associated with the article are available under the terms of the Creative Commons Zero "No rights reserved" data waiver (CC0 1.0 Public domain dedication). 

As a law enforcement body, we are bound to official secrecy. Scrutinising and anonymising data was only done within an acceptable effort. Considering these restrictions, we provide raw data for all experiments presented in our work ( Figure 1– Figure 4) and of the 18 most frequent pigments of the market surveys, as shown in Table 1. Further, we created an Excel sheet ( Dataset 4) with intermediary data of the market surveys.

F1000Research: Dataset 1. MALDI raw data are provided as MASTER_RUN-, CAL-, COR-, LBL-, RAW, RUN, STATS- and UAP- files using Launchpad™ version 2.9.3 software (Shimadzu-Biotech Corp., Kyoto). The data include the raw high resolution spectra of Figure 1, Figure 3 and Figure 4, 10.5256/f1000research.13035.d184766 ^19^

F1000Research: Dataset 2. MALDI raw data are provided as TXT-files in ASCII-format using Launchpad™ version 2.9.3 software (Shimadzu-Biotech Corp., Kyoto). The data include the high resolution raw spectra of Figure 1, Figure 3 and Figure 4, 10.5256/f1000research.13035.d184767 ^20^

F1000Research: Dataset 3. NIST spectrum peak lists are provided as MSP-files in ASCII-format with a mass resolution of 1 D (msp) using the software of NIST 2.0 (USA). The data include the NIST spectra of Figure 2, Table 1 and method validation (HPLC verification), 10.5256/f1000research.13035.d184768 ^21^

F1000Research: Dataset 4. Intermediary data of Table 1 and a list of suppliers of pigments of Table 1 are provided as Excel-files (XLS). The data include sample-no, declaration of prohibited and legal pigments and reversed match values of pigments by NIST-software (Rfit), 10.5256/f1000research.13035.d184769 ^22^

F1000Research: Dataset 5. An example of the construction of our home-made spectrum library using the NIST software 2.0 (USA) is shown as JPG-file. The data include compound name, structural formula, molecular mass, CAS-no., analysis date, intensity of the main spectrum peaks and synonyms, 10.5256/f1000research.13035.d184770 ^23^
