# Supplementary material for: Identification of organic pigments in tattoo inks and permanent make-up using laser desorption ionisation mass spectrometry
Source: F1000Res. 2018 Jan 8;6:2034. Originally published 2017 Nov 21. [Version 2] doi: 10.12688/f1000research.13035.2 (PMC5728192; doi:10.12688/f1000research.13035.2)
Supplement: Peer review history

# Peer review: Referee response for version 2

**DOI:** 10.5256/f1000research.14747.r29570

**Published:** 2018-01-24

**Authors:** Guido Vogel

**Affiliations:** 1 Mabritec AG, Riehen, Switzerland

I approve the revised version 2 of the article of Niederer *et al*.

I have read this submission. I believe that I have an appropriate level of expertise to confirm that it is of an acceptable scientific standard.

# Peer review: Referee response for version 2

**DOI:** 10.5256/f1000research.14747.r29571

**Published:** 2018-01-08

**Authors:** Christoph Hutzler, Ines Schreiver

**Affiliations:** 1 Department for Chemical and Product Safety, German Federal Institute for Risk Assessment (BfR), Berlin, Germany

No further comments necessary.

We have read this submission. We believe that we have an appropriate level of expertise to confirm that it is of an acceptable scientific standard.

# Peer review: Referee response for version 1

**DOI:** 10.5256/f1000research.14134.r28228

**Published:** 2017-12-11

**Authors:** Christoph Hutzler, Ines Schreiver

**Affiliations:** 1 Department for Chemical and Product Safety, German Federal Institute for Risk Assessment (BfR), Berlin, Germany

The method article from Niederer et al. describes the identification of organic pigments in the complexes matrices of tattoo ink and permanent make-up using Matrix assisted laser desorption/ionization (MALDI) mass spectrometry. The authors created a reference library of >150 colorants and analyzed a total of 451 inks. The qualitative method described in this article was validated and counter checked with a complementary HPLC analysis suitable for soluble colorants.

 The method is highly valuable to identify tattoo pigments in the course of market surveys. Their results show, that false declaration and fraud are frequent among tattoo and permanent make-up inks.

 In the following minor amendments are suggested:

- Despite the common use of the plural form in the abbreviation PMUs, make-up itself comes without a plural and is considered uncountable.
- The authors used both the color index and names of pigments to facilitate readability for scientists from different fields. In the section market survey an additional abbreviation PG7 appears. This might be replaced by either of the other identifiers.
- In the abstract and section "market survey" the authors refer to the ban of pigments because of the hazardous properties and toxicological concerns. Although this might be correct for some pigments, the ban for tattoo inks might also derive from missing toxicological data instead of data claiming hazards, e.g. in the case of pigment green 7.

In summary, the presented method is of major value not only in the field of tattoo and permanent make up compliance, but may also be applied to cosmetic products or used in forensic analyses.

We have read this submission. We believe that we have an appropriate level of expertise to confirm that it is of an acceptable scientific standard.

# Response

**Published:** 2017-12-20

**Authors:** Markus Niederer

**Affiliations:** State Laboratory Basel-City, Switzerland

We thank the reviewers for their constructive comments regarding our study.

1. As requested, the plural of make-up was corrected.
2. In the section “market survey” the abbreviation PG7 was replaced by Pigment Green 7.
3. In the abstract and section "market survey" text passages regarding the ban of pigments were modified.

# Peer review: Referee response for version 1

**DOI:** 10.5256/f1000research.14134.r28230

**Published:** 2017-12-08

**Authors:** Guido Vogel

**Affiliations:** 1 Mabritec AG, Riehen, Switzerland

The article by Niederer et al. on the "Identification of organic pigments in tattoo inks and permanent make-ups using MALDI TOF MS“ is very well written and discusses a new approach for the detection of potentially dangerous organic pigments in tattoo inks.

 The new method applying LDI- and MALDI-TOF MS is a big improvement as compared to those applied so far and will be of great help to authorities in their responsibility to regulate the use these pigments in tattoo studios. The importance of the availability of such methods is underlined by the very strong increase of persons with tattoos or permanent make-up in the last years.

 The applicability of this new identification method was clearly demonstrated in the many years of testing it with real life samples.

 I do not have any suggestions for improving the text and the figures. They are perfect in my opinion the way they are.

 Only the title could be improved by stating the use of LDI-TOF MS next to MALDI-TOF MS.

I have read this submission. I believe that I have an appropriate level of expertise to confirm that it is of an acceptable scientific standard.

# Response

**Published:** 2017-12-20

**Authors:** Markus Niederer

**Affiliations:** State Laboratory Basel-City, Switzerland

We would like to thank the reviewer for his time and constructive feedback.

 As requested the title and some text passages were changed to clarify that LDI- instead of MALDI-TOF-MS was used in most cases.
